# *nkx2.1* and* nkx2.4* genes function partially redundant during development of the zebrafish hypothalamus, preoptic region, and pallidum

**DOI:** 10.3389/fnana.2014.00145

**Published:** 2014-12-02

**Authors:** Martha Manoli, Wolfgang Driever

**Affiliations:** ^1^Developmental Biology, Faculty of Biology, Institute Biology I, University of FreiburgFreiburg, Germany; ^2^Centre for Biological Signaling Studies (BIOSS), University of FreiburgFreiburg, Germany

**Keywords:** hypothalamus, preoptic region, pallidum, diencephalon, telencephalon, neural patterning, neuroendocrine neurons, zebrafish

## Abstract

During ventral forebrain development, orthologs of the homeodomain transcription factor Nkx2.1 control patterning of hypothalamus, preoptic region, and ventral telencephalon. However, the relative contributions of Nkx2.1 and Nkx2.4 to prosencephalon development are poorly understood. Therefore, we analyzed functions of the previously uncharacterized *nkx2.4*-like *zgc:171531* as well as of the presumed *nkx2.1* orthologs *nkx2.1a* and *nkx2.1b* in zebrafish forebrain development. Our results show that *zgc:171531* and *nkx2.1a* display overlapping expression patterns and a high sequence similarity. Together with a high degree of synteny conservation, these findings indicate that both these genes indeed are paralogs of *nkx2.4*. As a result, we name *zgc:171531* now *nkx2.4a*, and changed the name of *nkx2.1a* to *nkx2.4b*, and of *nkx2.1b* to *nkx2.1*. In *nkx2.1, nkx2.4a*, and *nkx2.4b* triple morpholino knockdown (nkx2TKD) embryos we observed a loss of the rostral part of prosomere 3 and its derivative posterior tubercular and hypothalamic structures. Furthermore, there was a loss of rostral and intermediate hypothalamus, while a residual preoptic region still develops. The reduction of the ventral diencephalon was accompanied by a ventral expansion of the dorsally expressed *pax6*, revealing a dorsalization of the basal hypothalamus. Within the telencephalon we observed a loss of pallidal markers, while striatum and pallium are forming. At the neuronal level, nkx2TKD morphants lacked several neurosecretory neuron types, including avp, *crh*, and *pomc* expressing cells in the hypothalamus, but still form *oxt* neurons in the preoptic region. Our data reveals that, while *nkx2.1, nkx2.4a*, and *nkx2.4b* genes act partially redundant in hypothalamic development, *nkx2.1* is specifically involved in the development of rostral ventral forebrain including the pallidum and preoptic regions, whereas *nkx2.4a* and *nkx2.4b* control the intermediate and caudal hypothalamus.

## Introduction

Regional specification of the central nervous system (CNS) commences during gastrulation and at neural plate stage, when patterning along the anteroposterior as well as the dorsoventral axes is initiated. The anteroposterior patterning assigns rostrocaudal regional identities (Lumsden and Krumlauf, 1996), and together with the dorsoventral patterning influences cell fate specification and consequently the generation of different neuronal subtypes (Tanabe and Jessell, 1996). Several *Nkx* genes act during patterning of the ventral CNS, and contribute to a molecular code for neuronal differentiation (Shimamura et al., 1995; Ericson et al., 1997; Pabst et al., 1998). Nkx2.1 is a member of the vertebrate Nkx homeobox transcription factor family (Pera and Kessel, 1998; Small et al., 2000; van den Akker et al., 2008). It is also known as thyroid transcription factor 1 (TTF-1) or Thyroid-specific enhancer-binding protein (T/ebp) because of its involvement in thyroid development (Guazzi et al., 1990; Mizuno et al., 1991; Elsalini et al., 2003). Two *nkx2.1* genes in the zebrafish genome have been previously described as paralogs*, nkx2.1a* and *nkx2.1b* (Rohr et al., 2001). Expression of *nkx2.1a* and *b* in the zebrafish CNS was reported to initiate toward the end of gastrulation in a rostrocaudal stripe in the medial anterior neural plate, giving rise to hypothalamus, preoptic region and ventral telencephalon (Rohr et al., 2001). In the medial neural plate and ventral neural tube, expression of mammalian *nkx2.1* has distinct anteroposterior and dorsoventral boundaries (Shimamura et al., 1995; Briscoe et al., 2000; Puelles and Rubenstein, 2003). *nkx2.1a* and *b* expression domains display a common posterior border, which resides in the basal part of prosomere 3 located ventrally to the prethalamus. Both are expressed in the posterior tuberculum and basal hypothalamus. However, the preoptic region, the basal telencephalon around the anterior commissure, and the alar preoptic region exclusively express *nkx2.1b* (Rohr et al., 2001; Lauter et al., 2013). The medial hypothalamus is characterized by *nkx2.1a* expression and low or absent *nkx2.1b* expression.

In mice, development of ventral hypothalamus and telencephalic medial ganglionic eminence depend on NKX2.1. In homozygous *Nkx2.1* knockout mice, ventral forebrain developmental abnormalities start anteriorly in the septal area and extend to the mammillary body of the hypothalamus (Kimura et al., 1996). Loss of NKX2.1 also causes a transformation of medial ganglionic eminence into lateral ganglionic eminence structures (Sussel et al., 1999). In addition, both in mouse and Xenopus embryogenesis removal of *nkx2.1* causes a dorsalization of the basal plate forebrain (Sussel et al., 1999; van den Akker et al., 2008). *Nkx* genes are also involved in neuronal differentiation. In the spinal cord the combinatorial expression of *Nkx* transcription factors (*Nkx6.1, Nkx2.2*, and *Nkx2.9*) specifies ventral identity of neurons (Briscoe et al., 2000; Sander et al., 2000). Less is known about potential involvements of Nkx2 factors in forebrain neuronal differentiation, where Nkx2.1 has been shown to contribute to cortical interneuron subtype specification (Butt et al., 2008).

Here, we report the identification, expression and functional analysis of the zebrafish *nkx2.4a* gene. During embryogenesis *nkx2.4a* is expressed in the hypothalamus in a manner similar to the gene previously reported as *nkx2.1a*. Phylogenetic analysis suggests that *nkx2.4a* resulted from an *nkx2.1* gene duplication event that is not restricted to teleosts (Price, 1993; Small et al., 2000; Wang et al., 2000). In zebrafish, like in mouse (Marín et al., 2002), *nkx2.4a* is expressed in a restricted area of the hypothalamus. In contrast to *nkx2.1*, *nkx2.4a* is not expressed in the telencephalon. Synteny and phylogenetic analysis reveals that the previously reported *nkx2.1a* is in fact a paralog of *nkx2.4*, and not of *nkx2.1*, and thus will be named *nkx2.4b* here in accordance with zebrafish nomenclature. Combined inactivation of *nkx2.1, nkx2.4a*, and *nkx2.4b* function by triple Morpholino knockdown (here called nkx2TKD) revealed their contribution to neural patterning and neuronal differentiation. In contrast to single knockdown, the ventral diencephalon of nkx2TKD morphants is dorsalized, revealing that *nkx2.1*, *nkx2.4a*, and *nkx2.4b* act redundantly in hypothalamic patterning. All three genes are required for development of specific subsets of neurosecretory neurons in the hypothalamus.

## Materials and methods

### Zebrafish husbandry

Zebrafish breeding and maintenance were carried out under standard conditions at 28.5°C (Westerfield, 2000). We used AB-TL wildtype zebrafish, the enhancer trap *ETvmat2:GFP* (Wen et al., 2008), and *smo*^*b*641^ (Barresi et al., 2000), which were identified morphologically. To inhibit pigmentation, embryos were incubated in egg water containing 0.2 mM 1-phenyl-2-thiourea. Embryos were staged according to Kimmel et al. (1995).

### Injection of morpholinos and RNA

The following morpholinos were used (Gene Tools LLC): *nkx2.4a* TBMO (ATG): 5′-GCTCAGCGACATGGTTCAGCCCGCA-3′, *nkx2.4a* SBMO (e1i1): 5′-TGCATTAGAAGAACTTACTTGTTGA-3′. The standard control SCMO, p53 ATG (Robu et al., 2007), *nkx2.1* (previously published as *nkx2.1b*) and *nkx2.4b* (published as *nk2.1a-1*) ATG morpholinos have been described (Elsalini et al., 2003). Morpholinos were diluted in H_2_O containing 0.05% phenol red or 0.05% rhodamine dextran. *nkx* morpholinos were injected at the one cell stage in different combinations at a total amount of 7.5 ng per embryo, plus an additional 2.5 ng p53 MO. As controls, 7.5 ng standard control morpholino (SCMO) and 2.5 ng p53 MO per embryo were injected.

The efficiency of the *nkx2.4a-e1i1*-SBMO was verified by RT-PCR using cDNA synthesized from 1, 2, and 3 dpf embryos injected with SCMO or *nkx2.4a-e1i1*-SBMO+*nkx2.4b*-MO+*nkx2.1*-MO and *p53*-MO at 2.5 ng each (same conditions as used for all nkx2TKD; **Figure 3B**). The PCR products where sequenced using primers spanning the whole *nkx2.4a* coding region: *nkx2.4a* F: 5′-ATGTCGCTGAGCCCAAAG-3′, *nkx2.4a* R: 5′-CTACCACGTTCTGCCATAAAGC-3′.

To analyse the specificity and efficacy of the translation blocking MOs (TBMOs) directed against the translation start sites of *nkx2.1*, *nkx2.4a*, or *nkx2.4b*, pCS2+-gfp-reporter plasmids were created which each harbor the respective morpholino target sequence, fused in frame to the GFP ORF. *gfp*-reporter mRNAs were generated from plasmids pCS2+-5′UTR-nkx2.4b-gfp, pCS2+-5′UTR-nkx2.1-gfp and pCS2+-5′UTR-nkx2.4a-gfp linearized with NotI, and transcribed using the SP6 mMessage mMachine kit (Ambion). *gfp*-reporter mRNAs were co-injected into one-cell stage embryos in combination with SCMO or the respective specific targeting morpholino. At epiboly stages, embryos were assayed for GFP fluorescence (**Figure 4**). We also attempted to validate the knockdown phenotypes by rescue experiments injecting mRNAs encoding the Nkx2.1 and Nkx2.4, however broad overexpression of Nkx2.1 or Nkx2.4 from injected mRNAs caused severely abnormal development likely because of gain-of-function effects due to ectopic expression (data not shown).

To verify the results obtained with the *nkx2.4a* splice blocking MO, we repeated the nkx2TKD substituting the *nkx2.4a* SBMO with the translation blocking *nkx2.4a* TBMO in the nkx2TKD mix, and analyzed *tyrosine hydroxylase* (*th*), *lhx5*, and *lhx6* expression (**Figure 6**). Both *nkx2.4a* SBMO or TBMO at 2.5 ng per embryo in combination with *nkx2.4b* and *nkx2.1* TBMO generated a similar phenotype.

Synthetic *lefty1* mRNA (mMessage mMachine kit, Ambion) was injected as described (Barth and Wilson, 1995; Thisse and Thisse, 1999).

### *In situ* hybridization and tunel staining

Whole-mount *in situ* hybridization (WISH) (Lauter et al., 2011) and fluorescent WISH with immunohistochemistry (Filippi et al., 2010) were performed as described. The following digoxigenin-labeled riboprobes were synthesized: *dbx1a* (Fjose et al., 1994), *dlx5a* (Akimenko et al., 1994), *foxa2* (Strähle et al., 1993), *lhx5* and *lhx6* (Toyama et al., 1995), *shha* (Ekker et al., 1995), *otpa* (Del Giacco et al., 2006; Ryu et al., 2007), *pax6* (Krauss et al., 1991b), *pomca* (Herzog et al., 2003), *nkx2.2* (Barth and Wilson, 1995). For generation of *nkx2.4b* (RefSeq NM_131589.1), *nkx2.1(b)* (NM_131776.1), and *nkx2.4a* (NM_001111166.1) probes, the coding sequences were PCR amplified and cloned into the TOPO PCRII vector using the primers *nkx2.4b* F: 5′-ATGTCCTTGAGCCCCAAAC-3′; *nkx2.4b* R: 5′-TCACCATGTTCTGCCGTACA-3′; *nkx2.1* F: 5′-ATGTCGATGAGCCCTAAGCA-3′; *nkx2.1* R: 5′-TCACCACGTCCTGCCATA-3′ (for *nkx2.4a* see above).

TUNEL assay was performed using the Apoptag *in situ* apoptosis detection kit (Chemicon) (Ryu et al., 2005). Classification of signal: “absent”—no blue stained cell detected in specific brain region; “few cells”—small number of blue stained cell (1–10) dispersed in specific brain region (example telencephalic region in **Figures 3I,J**); “many cells” accumulation of >10 blue stained cells, often clustered, in specific brain region.

### Microscopy and image analysis

Transmitted light images were acquired using a Zeiss Axioskop compound microscope. Fluorescently labeled embryos were documented by confocal image stacks using a Zeiss LSM510. Images shown are z-projections of defined sets of consecutive focal planes assembled with the Zeiss Zen 2012 software.

### Sequence alignments and synteny

NKX protein sequences were aligned and analyzed with CLC Genomics Workbench 5 (www.clcbio.com) using the distance based method to generate a phylogenetic tree. The NCBI accession numbers are: NK2.4b [Danio rerio] NP_571664.1, NK2.1(b) [Danio rerio] NP_571851.1, Nkx-2.1 [Mus musculus] NP_001139670.1, Nkx-2.1 isoform 2 [Homo sapiens] NP_003308.1, Nkx-2.1 isoform 1 [Xenopus tropicalis] XP_002935383.1, Nkx-2.4a [Danio rerio] NP_001104636.1, Nkx-2.4 [Mus musculus] NP_075993.1, Nkx-2.4 [Homo sapiens] NP_149416.1, Nkx-2.4 [Xenopus tropicalis] XP_002939478.1, Nkx-2.2 [Homo sapiens] NP_002500.1, Nkx2-2 protein [Mus musculus] AAI38160.1, Nkx-2.2 isoform X1 [Xenopus tropicalis] XP_002939477.1, Nkx-2.2a [Danio rerio] NP_571497.1, Nkx-2.3 [Homo sapiens] NP_660328.2, Nkx2-3 [Mus musculus] CAA72002.1, Nkx-2.3 [Xenopus tropicalis] XP_002937234.1, Nkx2.3 [Danio rerio] AAC05228.1, Nkx-2.5 [Mus musculus] NP_032726.1, Nkx-2.5 isoform 1 [Homo sapiens] NP_004378.1, Nkx2.5 [Danio rerio] AAC05229.1, Nkx2-5 [Xenopus tropicalis] AAI60531.1

Synteny between the mouse and zebrafish *nkx2* genes was visualized using Cinteny (http://cinteny.cchmc.org).

## Results

### *Nkx2.1* and *Nkx2.4* genes in zebrafish

**Figure 1:**
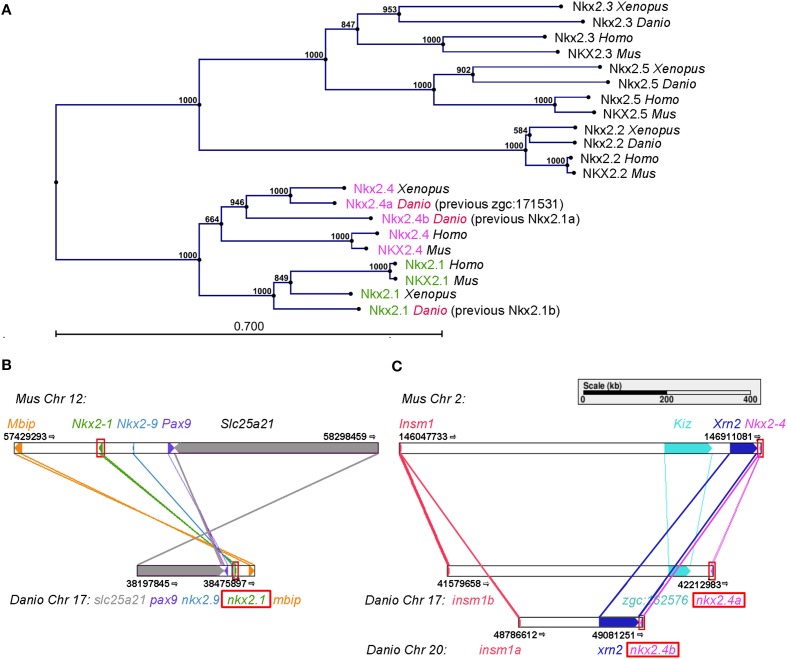
**Positioning *nkx2.1* and *nkx2.4* within the *nkx2* homeobox transcription factor family. (A)** Phylogenetic tree of five members of the Nkx2 homeobox transcription factor family in zebrafish, Xenopus, mouse, and human. Distance based tree calculations used full-length protein sequences (Materials and Methods). The numbers at the nodes are bootstrap confidence levels from 1000 replicates. The scale bar represents 0.7 substitutions per position. **(B,C)** Genetic synteny at the *nkx2.1* **(B)** and *nkx2.4* **(C)** loci in mouse and zebrafish analyzed using Cinteny (cinteny.cchmc.org). The arrows indicate transcriptional direction.

We systematically searched for zebrafish genes that may interact and be co-expressed with *nkx2.1* transcription factors during ventral diencephalon development, and noticed that the *zgc:171531* gene (ENSDARG00000075107) encodes an NKX2 family member. *zgc:171531* has recently been reported to be expressed in the hypothalamus during late somitogenesis stages (Armant et al., 2013), but its function has not been studied. We analyzed the phylogenetic relationship of Zgc:171531 to other members of the Nkx protein family, and could confirm the suggestion by Armant et al. (2013) that Zgc:171531 is an ortholog of mouse NKX2.4 (Figure 1). Phylogenetic tree analysis showed that zebrafish Zgc:171531 is the closest relative of mammalian NKX2.4, and that Zgc:171531and Nkx2.1 are more closely related to each other than to any other Nkx2 transcription factor family member (Figure 1A). Therefore, we refer to Zgc:171531 as Nkx2.4a.

To explore the evolutionary history of the *nkx2.1* and *nkx2.4* genes, we searched for synteny conservation. We found that the gene previously reported as zebrafish *nkx2.1b* is located in a microsyntenic chromosomal region related to mouse *Nkx2.1* (Figure 1B), thus *nkx2.1b* is indeed the ortholog of mouse *Nkx2.1*. Zebrafish *zgc:171531* resides in a microsyntenic chromosomal region related to mouse *Nkx2.4*. Importantly, also zebrafish *nkx2.4b* (previously named *nkx2.1a*) shares synteny with mouse *Nkx2.4* (Figure 1C). Thus, we conclude that *nkx2.4a* and *nkx2.4b* are indeed paralogous NKX2.4 genes derived from the teleost specific genome duplication. In contrast, the previous *nkx2.1a* and *nkx2.1b* are not paralogs, and *zgc:171531* is now named *nkx2.4a*, while *nkx2.1a* is named *nkx2.4b*, and *nkx2.1b* is named *nkx2.1*. This change in nomenclature was approved by the zebrafish gene nomenclature committee (see www.zfin.org).

**Figure 2:**
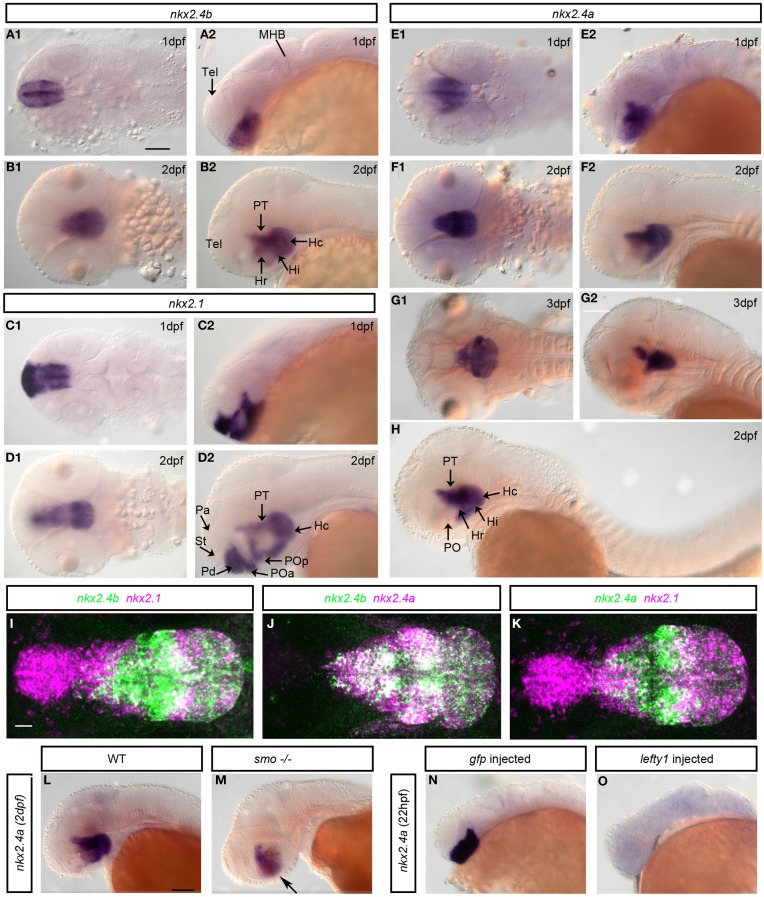
**Expression and regulation of *nkx2.1, nkx2.4a*, and *nkx2.4b*. (A,B)** *nkx2.4b*, **(C,D)** *nkx2.1* and **(E–H)** *nkx2.4a* mRNA expression analyzed by whole mount *in situ* hybridization at indicated stages. **(I–K)** Expression of *nkx2.4a* and *nkx2.4b* and *nkx2.1* were detected by double fluorescent whole mount *in situ* hybridization of 2 dpf embryos, magenta and green channels as indicated in panel headings. Shown are 140–190 μm Z-projections of confocal image stacks. **(L,M)** Expression of *nkx2.4a* in WT and *smo* homozygous mutant embryo. Arrow: residual *nkx2.4a* expressions (**M**, *n* = 10). *nkx2.4a* expression in controls **(N)** and embryos with Nodal signaling inactivated by *lefty1* mRNA injection (**O**: 65% of embryos showed a complete loss, 25% a reduction, 10% normal *nkx2.4a* expression; *n* = 25). **(A1–G1,L-O)** lateral views; **(A2–G2,I–K)** dorsal views. Anterior to left. Scale bar in **A1** = 100 μm (for **A–H**), in **I** = 20 μm (for **I–K**), in **L** = 100 μm (for **L–O**).

We analyzed expression of *nkx2.4a* in comparison to *nkx2.1* (Figure 2) (Rohr et al., 2001; Tessmar-Raible et al., 2007). At 1 dpf *nkx2.4a* is expressed within the forebrain in basal prosomere 3 including the hypothalamus and the posterior tuberculum, and with decreasing expression levels into the preoptic hypothalamic areas of the basal secondary prosencephalon (Figure 2E). In contrast to *nkx2.1* (Figures 2C,D), expression of *nkx2.4a* was not detected in the alar hypothalamic region (anterior preoptic region) or the pallidum of the secondary prosencephalon. At 2 dpf *nkx2.4a* expression is downregulated in the basal hypothalamic preoptic area, but continues in the posterior tuberculum and caudal hypothalamus (Figure 2F). At 3 dpf *nkx2.4a* expression persists in the posterior tuberculum and hypothalamus (Figure 2G), similar to *nkx2.4b* (Figures 2A,B). This expression pattern is maintained until 4 dpf (data not shown). Thus, all three *nkx2* genes are expressed in the posterior tuberculum as well as in the basal hypothalamus. However, *nkx2.1* is also expressed in the basal telencephalon around the anterior commissure as well as in the alar hypothalamus preoptic region. *nkx2.4a*, in contrast to *nkx2.4b* (Elsalini et al., 2003), is not expressed in the thyroid gland (Figure 2H). Double fluorescent WISH of *nkx2.4a* expression in combination with *nkx2.4b* or *nkx2.1* revealed broad coexpression of *nkx2.4a* and *nkx2.4b* (Figure 2J), whereas *nkx2.4a* and *nkx2.1* coexpression is restricted mainly to the intermediate hypothalamus (Figures 2I,K).

### Nodal and Shh signaling control *Nkx2.4a* expression

Nodal signaling has been revealed essential for initiation, and Shh for maintenance of *nkx2.1* and *nkx2.4b* expression (Rohr et al., 2001). Smoothened is a key transmembrane effector essential for transmitting Hedgehog signals into the cell (Rohatgi and Scott, 2007). *smoothened homolog (smo^−/−^)* zygotic mutant embryos have severely reduced Shh signaling, although no complete loss occurs due to residual maternal Smo activity (Barresi et al., 2000; Rohr et al., 2001). We detected *nkx2.4a* expression at reduced levels in *smo^−/−^* embryos (Figures 2L,M), indicating that Hedgehog signaling activity is required to maintain *nkx2.4a* expression. However, Shh is not required for *nkx2.4a* initial induction, similar to what has been shown for *nkx2.4b*. We then overexpressed *lefty1* mRNA, encoding an inhibitor of Nodal activity, which resulted in complete loss of *nk2.4* expression in >50% of experimental embryos (Figures 2N,O). Thus, Nodal is strictly required to induce *nkx2.4a* expression, while Shh contributes to its maintenance.

### *Nkx2.1* and *Nkx2.4a/4b* combined knockdown severely affects the hypothalamus

**Figure 3:**
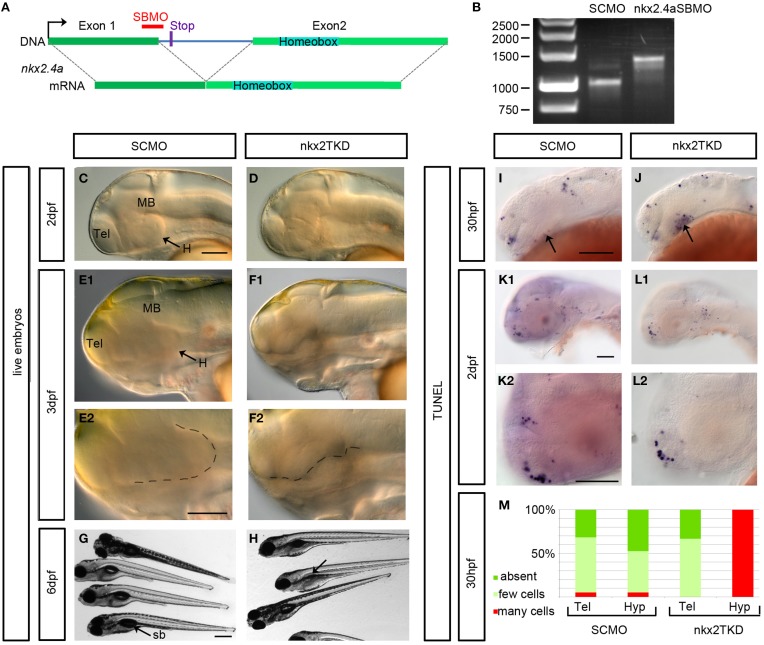
**Phenotype of *nkx2.1, nkx2.4a, nkx2.4b* triple (nkx2TKD) morphants. (A)** Schematic representation of the *nkx2.4a* gene with the morpholino binding site and the stop codon (12 bases into intron) terminating the protein when intron excision is blocked by the SBMO. **(B)** RT-PCR for *nkx2.4a mRNA* prepared from 3 dpf embryos injected with 7.5 ng SCMO or with 2.5 ng each of *nkx2.4a-e1i1*-MO, *nkx2.4b* and *nkx2.1* ATG MOs. The *nkx2.4a*-e1i1 MO effectively blocks the splice donor site at the exon1-intron1 boundary, giving rise to a longer cDNA. The mature *nkx2.4a* mRNA is nearly completely eliminated under these conditions. **(C–F)** Morphological *in vivo* phenotype of nkxTKD knockdown combined with p53MO knockdown documented using DIC transmitted light microscopy at indicated stages. **(E2–F2)** Close-ups with the ventral border of the diencephalon outlined by dotted lines. **(G,H)** nkxTKD larvae survived at least until 6 dpf (swim bladder is not inflated in fish in **H** indicated by an arrow). **(I–M)** TUNEL staining for apoptotic cells in 30 hpf and 2 dpf SCMO plus p53MO-injected control and nkxTKD plus p53MO morphant embryos (arrows in **I** and **J**: hypothalamus) (**K–L,K2,L2** show close-ups). **(M)** Quantification of number of TUNEL stained apoptotic cells in nkxTKD+p53 (*n* = 12) morphant larvae compared to SCMO+p53MO injected larvae (*n* = 19). Lateral views, anterior at left. Abbreviations see Table 1. Scale bars = 100 μm except **G**: scale bar = 400 μm.

**Figure 4:**
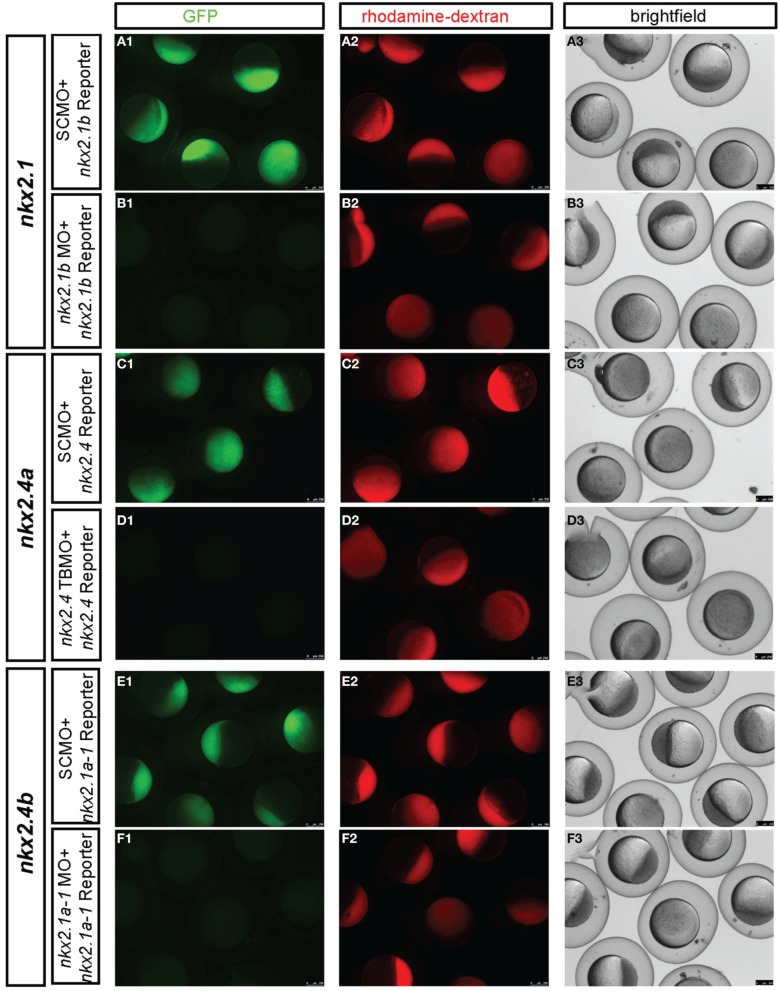
**Experimental validation of *in vivo* knockdown efficiency of translation blocking morpholinos used in this study**. MO knockdown efficiency was demonstrated by co-injection of GFP reporter mRNAs with the morpholino binding sites engineered at the start ATG of GFP. The following GFP reporter were injected at 100 pg mRNA per embryo: **(A,B)** *5′UTR-nkx2.1-gfp* mRNA, **(C,D)** *5′UTR-nkx2.4a-gfp* mRNA, and **(E,F)** *5′UTR-nkx2.4b-gfp* mRNA. Morpholinos used are indicated in boxes at left of each panel and were injected at 5 ng each, controls containing the same amount of SCMO. *nkx2.1* ATG MO and *nkx2.4b* ATG MO morpholinos have been described previously (Elsalini et al., 2003). All embryos were also co-injected with rhodamine-dextran, which was used to sort embryos after 4–5 h. of development for homogenous amounts and distribution of injected material. Knockdown efficiency was then documented between 4 and 5 hpf by pictures taking with the epifluorescence dissecting microscope in the red channel to verify that embryo were injected, in the green channel to control efficiency of GFP knockdown, and in the transmitted light channel to control morphology and viability of embryos. *nkx2.4b* and *nkx2.1* and *nkx2.4a*TBMO morphants were rhodamine-dextran fluorescence levels similar to control-injected embryos but showed no expression of the respective GFP reporters, indicating that these TBMOs effectively bind and block translation of target mRNAs *in vivo*.

To examine the role of Nkx2.4a we performed gene knockdown using antisense MOs. The *nkx2.4a* SBMO is complementary to the exon1-intron1 boundary (Figure 3A). We have amplified *nkx2.4a* mRNA by RT-PCR from *nkx2.4a* SBMO injected embryos, and found intron1 to be completely retained in the mature mRNA (Figure 3B). Sequencing of the splice-blocked mRNA revealed a premature stop codon truncating the Nkx2.4a protein before the homeodomain. To determine the efficiency of the *nkx2.4a* TBMO a *nkx2.4a-gfp* reporter mRNA with the MO binding site at the GFP ATG position was co-injected with the *nkx2.4a* TBMO and shown to completely suppress GFP expression (Figure 4). Thus, *nkx2.4a* SBMO and TBMO efficiently inhibit Nkx2.4a expression. Knocking down *nkx2.4a* alone did not result in any detectable morphological phenotype (data not shown).

**Table 1:** **Anatomical abbreviations**.

1	Basal prosomer 1
2	Basal prosomer 2
3c	Caudal portion of basal prosomer 3 (dbx1+)
3r	Rostral portion of basal prosomer 3 (Pt)
Arc	Arcuate nucleus
H	Hypothalamus
HB	Hindbrain
Hc	Caudal hypothalamus
Hi	Intermediate hypothalamus
Hr	Rostral hypothalamus
MB	Midbrain
OB	Olfactory bulb
P3c	Caudal Prosomere 3
Pa	Pallium
Pd	Pallidum
pit	Pituitary
PO	Preoptic region
POa	Anterior preoptic region
POp	Posterior preoptic region
Pr	Pretectum
PT	Posterior tuberculum
pTh	Prethalamus
sb	Swim bladder
SP	Subpallium
SPV	Supraoptoparaventricular region
St	Striatum
Tel	Telencephalon
Th	Thalamus
zli	Zona limitans intrathalamica

The similarity in amino acid sequences and the overlapping expression patterns suggest that *nkx2.1, nkx2.4a*, and *nkx2.4b* may act redundantly. Therefore, we aimed at combined knockdown of all three *nkx2* genes. We decided to use Morpholino antisense knockdown to study the combined activity of these three genes, because mutations in none of these genes are available, and even if mutations may become available at some point, the genetic analysis of triple mutations providing the desired phenotype only in one out of 64 embryos is cumbersome. ATG-morpholinos specific to both *nkx2.1* genes have been published (Elsalini et al., 2003). We confirmed their efficacy by knockdown of GFP expression from injected reporter mRNAs (Figure 4). *nkx2.4b* TBMO, *nkx2.1* TBMO, and *nkx2.4a* SBMO were co-injected at 2.5 ng each per embryo in combination with p53 morpholino (for exact amounts used in each figure panel see Supplemental Table 1). Given that some morpholinos cause cell death irrespective of knockdown of specific gene function (Robu et al., 2007), we included p53 MO in our nkx2TKD knockdown mix in all experiments. Live nkx2TKD embryos and larvae were analyzed for morphological abnormalities using transmitted light microscopy (Figures 3C–H). At 2 dpf nkx2TKD embryos developed a severe loss of hypothalamic structures, as well as abnormalities in the preoptic region (Figures 3C,D). At 3 dpf the massive loss of ventral forebrain tissue became more prominent (Figures 3E,F). With the exception of the ventral forebrain, nkx2TKD embryos and larvae developed anatomical structures morphologically similar to control WT embryos, and overall progress of development was not affected. nkx2TKD larvae established blood circulation and survived at least until 6 dpf (Figures 3G,H), but frequently did not fully inflate the swim bladder.

To test whether nkx2TKD embryos may develop apoptosis, we performed TUNEL assays. Apoptosis was clearly increased in the hypothalamus of morphants compared to SCMO injected embryos at 30 hpf (Figures 3I,J,M). However, at 2 dpf apoptosis levels in nkx2TKD morphants were similar to SCMO injected control larvae (Figures 3K,L). Thus, nkx2TKD causes increased apoptosis in those brain regions of *nkx2.1/2.4* expression specifically when the hypothalamus forms, but not at later stages.

### Knockdown of *Nkx2.1/4a/4b* severely affects ventral forebrain pattern formation

**Figure 5:**
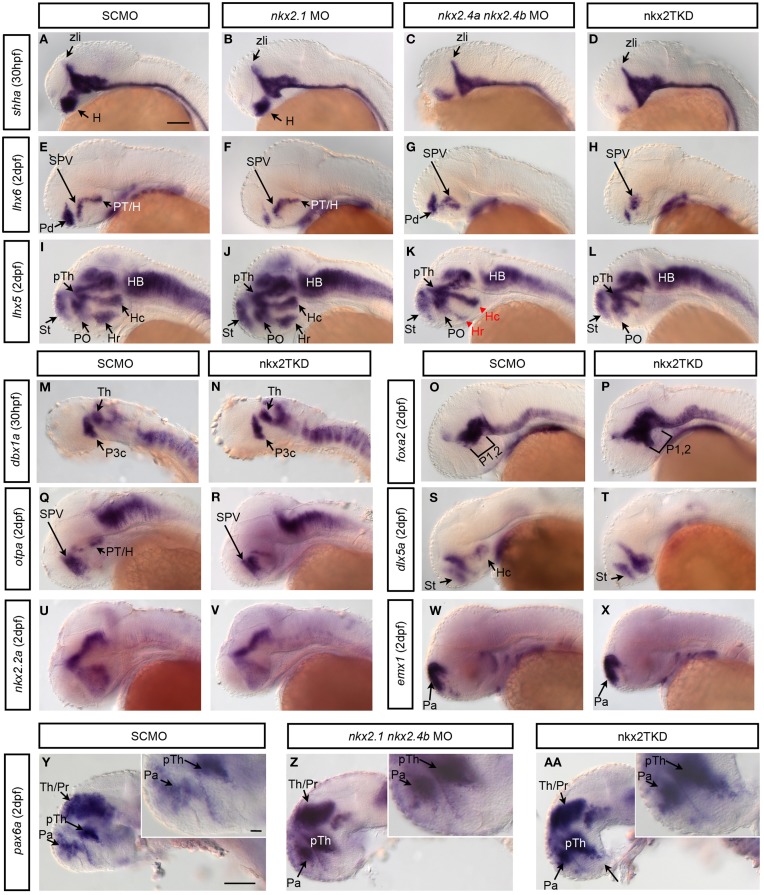
***nkx2.1*, *nkx2.4a*, and *nkx2.4b* knockdown affects forebrain patterning**. Zygotes were injected with single or combinations of Nkx2 gene morpholinos as indicated in boxes above the panels, and incubated until 30 hpf **(A–D,M,N)** or 2 dpf **(E–L,O–AA)**. Expression of patterning genes were analyzed by WISH. **(A–D)** Hypothalamic *shha* expression in **(B)** *nkx2.1* morphants is largely normal, with a minor reduction (*n* = 10.10); **(C)** *nkx2.4a/4b* double morphants (*n* = 10.10) is more severely reduced; **(D)** nkx2TKD embryos is severely reduced in the rostral forebrain (*n* = 29.29). **(E–H)** *lhx6*, and **(I–L)** *lhx5* expression in morphants at 2 dpf (**E**, *n* = 42.42; **F**, *n* = 11.11; **G**, *n* = 4.4; **H**, *n* = 67.67; **I**, *n* = 30.30; **J**, *n* = 7.7; **K**, *n* = 9.9; **L**, *n* = 23.23) (red arrowhead in **K** indicates affected area/reduced expression). (M,N) *dbx1a* expression was not perturbed by nkx2TKD at 30 hpf. **(O,P)** nkxTKD leads to a slight anterior expansion of *foxa2* expression in basal prosomeres 1 and 2 (**O**, *n* = 22.22; **P**, *n* = 11.11). **(Q,R)** *otpa* and **(S,T)** *dlx5a* expression were affected in the posterior tuberculum and hypothalamus of nkx2TKD morphants at 2 dpf (**Q**, *n* = 34.34; **R**, *n* = 60.60; **S**, *n* = 16.16; **T**, *n* = 12.12). **(U,V)** nkx2TKD affects the expression pattern of *nkx2.2* only in the caudal hypothalamus (*n* = 14.14). **(W,X)** The pallial telencephalic *emx1* expression is not affected in nkx2TKD. **(Y,AA)** In nkx2TKD morphants the prethalamic *pax6* expression domain expands into the ventral hypothalamus (inset in **AA**, *n* = 13.13), while double *nkx2.1* and *nkx2.4b* morphant have a less severe ventral *pax6* expansion (inset in **Z**, *n* = 25.25). Lateral views, anterior is to the left. Abbreviations see Table 1. lateral views. Black arrows point at positions of anatomical structures labeled by abbreviation at start of arrow. Scale bar = 100 μm in **(A)** for **(A–X)**, in **(Y)** for **(Y–AA)**.

Shh is a key determinant of ventral pattern formation in the neural plate. Studies have shown that its expression in the basal telencephalon depends on *nkx2.1* function in mice (Sussel et al., 1999). In wild-type zebrafish embryos *shha* (Figure 5A) is expressed in floor plate and underlying axial mesoderm, as well as in the zona limitans intrathalamica (ZLI; Scholpp et al., 2006), which is a narrow transverse region between the prethalamus and thalamus (Shimamura et al., 1995; Kiecker and Lumsden, 2004). In *nkx2.1* morphants *shha* expression was mostly normal, with only a minor reduction in the hypothalamus (Figure 5B). In *nkx2.4a/4b* double morphants (Figure 5C) we detected a more severe reduction of the hypothalamic *shha* expression. In nkx2TKD embryos *shha* expression in the hypothalamus was almost completely eliminated (Figure 5D), while in the ZLI and caudal to it *shha* expression appeared normal. Thus, all three *nkx2* genes redundantly regulate *shha* expression in the hypothalamus. Caudal to prosomer 3 *shha* expression was not affected in nkx2TKD morphants. This is consistent with the finding that the expression pattern of *foxa2*, a marker for medial and lateral floor plate caudal to prosomere 2 (Odenthal and Nusslein-Volhard, 1998), appeared unaffected in nkx2TKD morphants with some enlargement of basal prosomeres 1 and 2 (Figures 5O,P). With respect to basal prosomere 3, the *nkx2.1* expression extends only into the rostral half of prosomere 3, while *dbx1a* is expressed in the caudal part of basal prosomere 3 (Lauter et al., 2013). *dbx1a* expression was not affected in nkx2TKD morphants, indicating normal development of the caudal portion of prosomer 3 (Figures 5M,N). This finding demonstrates that brain regions caudal to the limit of *nkx2.1/4a/4b* expression are not affected upon nkx2TKD.

**Figure 6:**
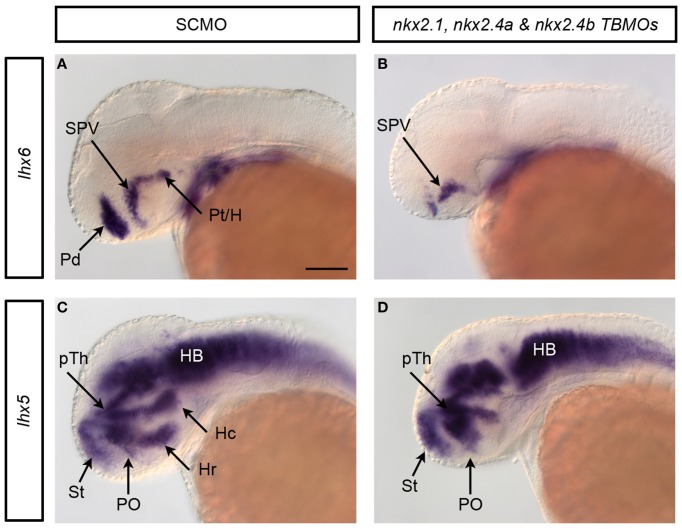
**Validation of nkx2TKD phenotype by independent *nkx2.4a* TBMO**. By designing a second, non-overlapping MO against *nkx2.4a* (*nkx2.4a* TBMO) concerns with off-target phenotypes can be addressed. The probability of both *nkx2.4a* MOs yielding a similar off-target phenotype is significantly lower compared to a single MO only (Eisen and Smith, 2008). The triple knockdown experiments with the *nkx2.4a* TBMO were analyzed for changes in *lhx6* **(A,B)** and *lhx5* **(C,D)** expression. The observed phenotypes were very similar to the nkx2TKD with the SBMO: the respective expression domains in the pallidum, the posterior tuberculum, the caudal and rostral hypothalamus were depleted in nkx2TKD with the *nkx2.4a* TBMO, while expression in other anatomical domains, including the alar preoptic region, was not significantly affected. Therefore, the analysis of nkx2TKD with the *nkx2.4a* TBMO confirmed the results obtained in our study with the nkx2TKD with the *nkx2.4a* SBMO. Black arrows point at positions of anatomical structures labeled by abbreviation at start of arrow.

A target of mouse NKX2.1 in the telencephalon is *Lhx6* (Sussel et al., 1999; Du et al., 2008). In our study, in *nkx2.1* morphants pallidal *lhx6* expression was strongly reduced, but not the diencephalic *lhx6* expression (Figures 5E,F). In *nkx2.4a* and *nkx2.4b* double morphants however pallidal *lhx6* expression was maintained, whereas in the diencephalon the caudal posterior tubercular expression was lost (Figure 5G). In nkx2TKD morphants, both the pallidal and posterior tubercular *lhx6* expression were severely reduced, while *lhx6* expression in the preoptic supraoptoparaventricular (SPV) region was retained (Figure 5H). These results were confirmed by analysing the more broadly expressed *lhx5* (Figure 5I). The *lhx5* domains in posterior tuberculum and caudal and rostral hypothalamus were depleted in nkx2TKD morphants, while expression in the alar preoptic region was still detectable (Figure 5L). In the telencephalon, the dorsal *lhx5* expression domain expanded ventrally into the pallidal area, from which *lhx5* was absent in wildtype. This ventral expansion of the telencephalic *lhx5* expression was also visible in *nkx2.1* morphants (Figure 5J), which otherwise had an unaltered *lhx5* expression pattern. Double *nkx2.4a* and *nkx2.4b* morphants did not develop the telencephalic *lhx5* expansion, but exhibited a reduction of the hypothalamic expression pattern (Figure 5K). The nkx2TKD expression changes of *lhx5* and *lhx6* were also validated in triple knockdown embryos using the *nkx2.4a* TBMO (Figure 6). The expression of *otpa* in the posterior tuberculum was strongly reduced in nkx2TKD morphants, while expression was still detected in the anterior preoptic area (Figures 5Q,R). Analysis of *dlx5a* reveals that the dorsal subpallium (striatum) still forms in nkx2TKD morphants (Figures 5S,T). In contrast, the caudal hypothalamic *dlx5a* domain was absent in triple morphants. Similar to what was observed for *lhx6* expression in nkx2TKD morphants, the *dlx5a* expression domain in the alar preoptic region appears expanded ventro-caudally (Figures 5S,T). *nkx2.2a* has a rostro-caudal domain extending approximately along the alar-basal boundary of the fore- and midbrain (Puelles and Rubenstein, 1993; Hauptmann and Gerster, 2000). This boundary remained unaffected in the triple morphants. Also, the prethalamic and thalamic as well as preoptic *nkx2.2a* expression domains appeared largely normal, while the caudal hypothalamic expression was reduced (Figures 5U,V). Analysing *emx1* expression, we did not observe any effect of nkx2TKD on the pallium (Figures 5W,X). In summary, these results show that loss of *nkx2.1/4a/4b* activity mainly affects three regions of the forebrain. In the caudal diencephalon, there is a loss of the rostral part of basal prosomer 3 and derivative posterior tubercular and hypothalamic structures, while the alar basal boundary appears non-shifted. Further rostrally, there is a loss of rostral and intermediate hypothalamic markers, while a residual preoptic region including the supraoptoparaventricular region develops in nkx2TKD morphants. Within the telencephalon, there is a loss of pallidal markers, while striatum and pallium are forming.

### Knockdown of *nkx2.1/4a/4b* leads to a dorsalization of the ventral diencephalon

Studies in mice suggested that *Nkx2.1* knockout causes a ventral expansion of dorsal diencephalic marker genes (Kimura et al., 1996). Therefore, we tested whether similar fate shifts occur in nkx2TKD embryos by analysing *pax6a* expression, which is predominantly expressed in dorsal parts of the diencephalon (Krauss et al., 1991a), as well as in cells at the pallial-subpallial boundary (Wullimann and Rink, 2001). In nkx2TKD embryos, *pax6* expression was expanded ventrally in its prethalamic domain, suggesting a loss of basal prosomere 3 (Figures 5Y–AA). Together with the data obtained for *dbx1a* expression (Figures 5M,N; caudal part of prethalamus and caudal part of basal prosomer 3; Lauter et al., 2013), it appeared that the rostral part of basal prosomere 3 was completely dorsalized, while the caudal *dbx1a* expressing part of prosomere 3 was not affected. Also, it appears that alar *pax6a* expression did not invade into the basal prosomere 1 and 2 regions, which in morphants appeared more pronounced caudal to prosomere 3 (see also 2 dpf *foxa2* expression Figures 5O,P). In contrast, when only *nkx2.1* and *nkx2.4b* were knocked down, no obvious ventral expansion of the *pax6a* domain into the hypothalamus was detectable. Thus, *nkx2.4a* can compensate loss of *nkx2.1* and *nkx2.4b* in hypothalamic patterning. In summary these results suggest that the loss of *nkx2.1/4a/4b* activity leads to a dorsalization of the basal prosomer 3 as well as of the pallidum.

### Nkx2.1/4a/4b control expression of *nkx2.1* and *nkx2.4* genes

**Figure 7:**
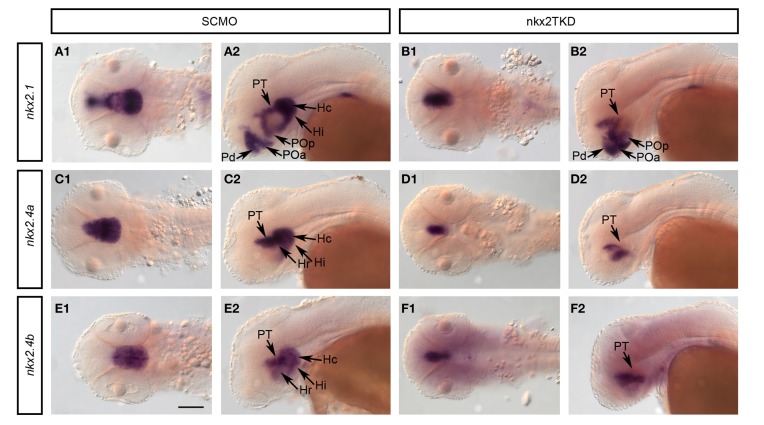
**Analysis of *nkx2* gene expression in *nkx2.1*, *nkx2.4a*, and *nkx2.4b* morphants**. Expression of *nkx2.1*, *nkx2.4a*, and *nkx2.4b* was analyzed in SCMO **(A,C,E)** and nkx2TKD morphants **(B,D,F)** at 2 dpf. **(A1–F2)** dorsal views, **(A2–F2)** lateral views; anterior is to the left. Abbreviations see Table 1. Black arrows point at positions of anatomical structures labeled by abbreviation at start of arrow. Scale bar = 100 μm in **A1** for all panels.

We next analyzed whether Nkx2.1/4a/4b activity is required for continued expression of these genes during embryonic development. Compared to controls, in nkx2TKD morphants the expression domains of all three genes were restricted to more medial forebrain regions, most pronounced for *nkx2.4a* and *nkx2.4b* (Figure 7). At 2 dpf, expression of all three genes was maintained in the posterior tubercular portion of the prosomer 3 derived basal hypothalamus in nkx2TKD embryos. In contrast, expression of all three *nkx2* genes was depleted in the intermediate and caudal hypothalamus. *nkx2.1/4a/4b* expression was reduced in the preoptic region. *nkx2.1* expression domains in the alar plate preoptic telencephalon and pallidum were maintained, albeit the size of the domain and the expression level appeared slightly reduced (Figures 7A,B). These results suggest that *nkx2.1* and *nkx2.4a/b* activity may be required to maintain their expression in specific domains of the developing forebrain. An alternate explanation may be that some hypothalamic tissue may get lost due to apoptosis (see Figures 3I,J).

### nkx2TKD affects hypothalamic neurosecretory populations

**Figure 8:**
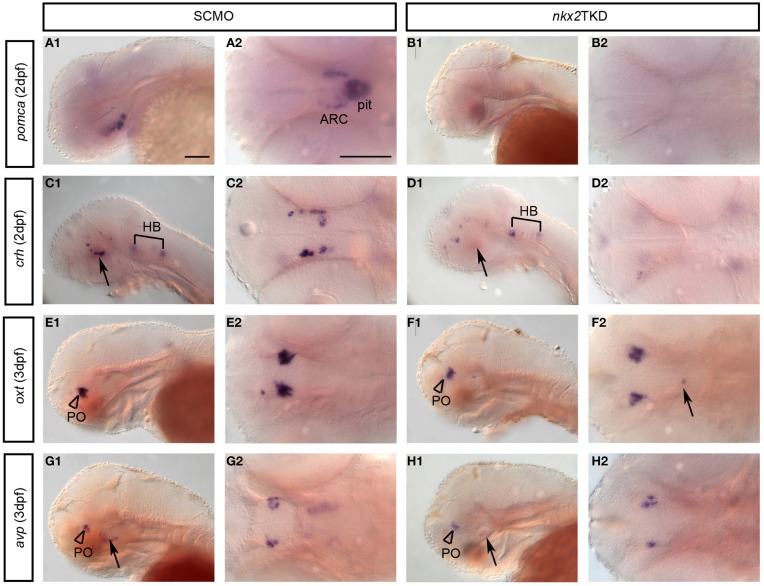
**Neuronal differentiation in the hypothalamus of nkx2TKD embryos**. Expression of differentiation markers for neuroendocrine neurons analyzed by WISH at 2, 3, and 4 dpf, stages as indicated. **(A,B)** Loss of *pomca* expression in the arcuate nucleus of the hypothalamus (arrowheads) and in the pituitary in nkx2TKD morphants. **(C,D)** *crh* neurons in the posterior tuberculum and hypothalamus (arrows) are reduced in nkx2TKD morphants (*n* = 7.7). **(E,F)** Formation of *oxt*-expressing cells in the PO (arrowheads) is not significantly affected in nkx2TKD morphants. However, in nkx2TKD morphants, *oxt*-expressing cells are detected at ectopic locations within the diencephalon (arrow) (*n* = 10.10). **(G,H)** In nkx2TKD morphants, *avp*-expressing cells in the PO (arrowheads) are still present, but lost in the hypothalamus (*n* = 9.9). **(A1–H1)** lateral views; **(A2–H2)** dorsal views, anterior to the left. The arrows point to the hypothalamus. Abbreviations see Table 1. Scale bars = 100 μm in **(A1)** for **(A1–J1)** and in **(A)** for **(A2–J2)**.

We next investigated which neuroendocrine systems were affected by loss of *nkx2.1* or *nkx2.4* activity. Expression of *pomca* (Hansen et al., 2003; Lohr and Hammerschmidt, 2011) is lost in the arcuate nucleus as well as the pituitary of nkx2TKD morphants (Figures 8A,B). To further characterize differential effects of nkx2TKD on ventral neuroendocrine vs. preoptic hypothalamus, we analyzed expression of the neuroendocrine hormone genes *corticotropin releasing hormone* (*crh), oxytocin (oxt)*, and *arginine vasopressin* (*avp)* at 2 and 3 dpf. *crh* expression (Chandrasekar et al., 2007) in the posterior tuberculum and hypothalamus of nkx2TKD morphants was completely absent (Figures 8C,D), while *crh* expressing neurons in all other regions developed normally. *oxt* is exclusively expressed in the preoptic region (Figures 8E,F), and similar to the preoptic domain of *avp* (Figures 8E,F) was not affected in triple morphants. In contrast, *avp* expression in the neuroendocrine ventral hypothalamus was strongly reduced or eliminated in nkx2TKD morphants (Figures 8G,H). We also occasionally observed *oxt*-expressing cells at ectopic locations posterior to the domain in the anterior hypothalamus in several nkx2TKD morphants (Figure 8F, arrow). In summary, nkx2TKD affects neuroendocrine development selectively in the hypothalamus, but not in the preoptic region.

## Discussion

The hypothalamus harbors many highly conserved neuroendocrine and neuromodulatory systems vital for the control of fundamental behavioral patterns and physiology. Hypothalamus organization and major developmental control centers have been well described (Puelles and Rubenstein, 2003; Moreno and Gonzalez, 2011). However, the complex organization and dynamic morphogenesis of the hypothalamus have hindered a more detailed understanding of molecular mechanisms controlling patterning and neuronal differentiation. *Nkx2.1* genes have a crucial role in development of the ventral forebrain, but analysis of mutant mice (Kimura et al., 1996; Sussel et al., 1999; Marín et al., 2002) and knockdown in *Xenopus* (van den Akker et al., 2008) have resulted in phenotypes that differentially affect the alar preoptic region and the basal hypothalamus. Here, we characterize a second *nkx2.1*-related gene in zebrafish, *nkx2.4a*. Partial redundancies between *nkx2.1* and *nkx2.4a*, which has not been previously knocked out in mice, may explain difficulties in understanding the role of NKX2.1 factors in ventral forebrain development.

*zgc:171531 / nkx2.4a* encodes a novel zebrafish Nkx2 homeobox transcription factor of high sequence similarity to Nkx2.1 and Nkx2.4. In light of synteny and phylogenetic tree analysis, the previously reported zebrafish *nkx2.1a* is more closely related to *nkx2.4a* than to *nkx2.1*. Recent phylogenetic analysis of rainbow trout Nkx2-4 also suggested zebrafish the previously *nkx2.1a* named gene to be a *nkx2.4* paralog (Uemae et al., 2014). Analysis of *nkx2.4a* expression in relation to the *nkx2.1* genes also reveals a strong similarity to the expression pattern in the brain of the gene previously reported as *nkx2.1a. nkx2.4a* is expressed in the posterior tuberculum and hypothalamus. *nkx2.4a* is not expressed in the preoptic region or pallidum, as *nkx2.1* is. For zebrafish, our data indicate that the gene previously named *nkx2.1a* is indeed a paralog of *nkx2.4*, and the two paralogous genes will be named *nkx2.4a* and *nkx2.4b* as orthologs of mammalian *Nkx2.4*, while *nkx2.1* is the only *Nkx2.1* ortholog in zebrafish.

Expression patterns similar to zebrafish have been reported for *nkx2.4* in *Xenopus* (Small et al., 2000; Ermakova et al., 2007). Mouse *Nkx2.4* expression (Price, 1993) is also restricted to the hypothalamus and absent from the preoptic region (Marín et al., 2002). Thus, it appears that the *nkx2.4* expression pattern is conserved throughout evolution, and based on the high sequence similarity in the homeodomain, may contribute to hypothalamic development in a redundant manner with *nkx2.1*. The two “parallel” *nkx2.1* and *nkx2.4* genes may also explain other riddles, for example *LhjTTF-1/LjNkx2.1* reported for *lampreta* to lack telencephalic expression (Ogasawara et al., 2001) may indeed be a *nkx2.4* ortholog.

Analysis of nkx2TKD embryos revealed that most *nkx2.1/4a/4b* expression in the basal hypothalamus depends on *nkx2.1/4a/4b* activity, except for the posterior tubercular domain. In contrast, it appears that the preoptic and pallidal domain do not strictly depend on *nkx2.1/4a/4b* activity. All three genes are reduced in the medio-lateral extent of their expression in nkx2TKD embryos, which would be in line with a reduced midline-derived activity like Shh required to establish and maintain these domains. Our findings are consistent with reports that *nkx2.1* expression is maintained in a smaller more medial-ventral domain in mice (Sussel et al., 1999), and in *Xenopus* (van den Akker et al., 2008) *nkx2.1* loss-of-function embryos. However, in mouse, expression of *nkx2.4* appears to strictly depend on *nkx2.1* activity (Marín et al., 2002), which would suggest changes in regulatory interactions in evolution.

**Figure 9:**
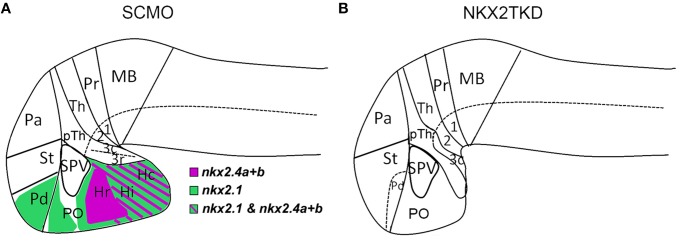
**Forebrain patterning abnormalities in nkx2TKD morphants at 2 dpf. (A,B)** Schematic sagittal representation of the anatomical domains in the brains of wildtype **(A)** and nkx2TKD morphant **(B)** embryos at 2 dpf. The expression domains of *nkx2.1*, *nkx2.4a*, and *nkx2.4b* are indicated in the wildtype scheme **(A)**, for color code see legend in figure. Abbreviations see Table 1.

While morpholino antisense oligonucleotide based knockdown of individual *nkx2.1/4* genes had only subtle effects on hypothalamus development, the combined knockdown of *nkx2.1, nkx2.4a* and *nkx2.4b* expression revealed that these genes together control specification and patterning of the basal hypothalamus. Figure 9 gives an overview of anatomical changes as revealed by marker gene analysis in nkx2TKD morphants. Triple morphant embryos were devoid of the basal hypothalamus including the rostral half of prosomere 3 and extending to the optic commissure. The defects in the nkx2TKD basal hypothalamus are thus limited to the region of *nkx2.1/4a/4b* expression, which extends caudally into the rostral half of basal prosomer 3 (Lauter et al., 2013). In nkx2TKD morphants, the caudal half of prosomer 3 expresses *dbx1a* normally, revealing that the *nkx2.1/4* activity has no effects caudal to its expression limits.

The loss of basal hypothalamic structures upon nkx2TKD is clearly revealed by loss or reduction of the expression domains of *lhx6*, *lhx5*, and *dlx5a* in this region. This basal plate tissue is dorsalized, as the prethalamic expression domain of *pax6a* expands to the ventral edge of the forebrain. However, the ventral expansion of dorsal fates does not reflect a global expansion of alar territories, as the dorsoventral positioning of the *nkx2.2* expression domain, which correlates with the alar-basal boundary, is not shifted. Thus, our data are consistent with the previously published dorsalization of ventral prethalamus in *nkx2.1* mutant mice (Sussel et al., 1999; Marín et al., 2002). They potentially explain discrepancies reported between mouse and *Xenopus* patterning mechanisms. The *xnkx2.1* knockdown has been reported to develop a much weaker basal hypothalamus phenotype (van den Akker et al., 2008) as compared to mice. This could be due to the fact that the *Xenopus nkx2.4* gene (Ermakova et al., 2007) could compensate loss of *nkx2.1*, while in mice *nkx2.4* expression depends on *nkx2.1* and thus the *nkx2.1* phenotype is much stronger.

In nkx2TKD morphants the preoptic region is less severely affected, and the SPV appears to develop normally. Both the basal hypothalamus and the preoptic region express *nkx2.1*, except for a small alar plate region of the SPV area characterized by *Orthopedia (otp)* expression (Puelles and Rubenstein, 2003). *otp* is also expressed in the posterior tuberculum and in the arcuate nucleus region. Interestingly, in nkx2TKD morphants the *otpa* expressing domain in the preoptic SPV area was not affected, while the posterior tubercular *otpa* domain was absent. This is consistent with *otpa* in the preoptic SPV not being co-expressed with *nkx2.1/4a/4b*, while in the posterior tuberculum *nkx2.1* is coexpressed (Ryu et al., 2007). The differentiation of neuroendocrine cells in the preoptic region has been studied in detail (Kurrasch et al., 2009; Machluf et al., 2011; Herget et al., 2014) and aids in dissecting the neuroanatomical structures effected in *nkx2.1* mutants. The less severe preoptic phenotype is in line with our finding that neuroendocrine cells develop in the preoptic region of nkx2TKD morphants. In contrast, development of essentially all tested basal hypothalamic neuroendocrine cells depends on *nkx2.1/4a/4b* activity.

The major subdivisions of the larval zebrafish telencephalon, pallium, striatum (dorsal part of area ventralis telencephali, Sdd) and pallidum (ventral part of area ventralis telencephali, Sdv), have been defined by marker gene expression including *lhx6* and *dlx2a* (Mueller et al., 2008; Ganz et al., 2011). Based on loss of *lhx6* expression, triple morphants have a severely reduced pallidum. Mammalian *lhx6* contains a highly conserved Nkx2.1 binding site in its promoter (Du et al., 2008), suggesting a direct and evolutionary conserved regulation. The ventral expansion of the striatal *lhx5* expression domain suggests that in the absence of *nkx2.1* activity, striatal fate expands into the pallidum, resulting in a dorsalization of the ventral telencephalon similar to that observed in *nkx2.1* mutant mice (Sussel et al., 1999; Marín et al., 2002). When we analyzed *emx* expression, we did not observed an expansion of the pallium, again similar to *nkx2.1* mutant mice (Sussel et al., 1999; Marín et al., 2002), but distinct from reports for *nkx2.1* knockdown in Xenopus (van den Akker et al., 2008).

In summary, our data reveals that *nkx2.1*, *nkx2.4a*, and *nkx2.4b* genes act partially redundant in zebrafish hypothalamic development. *nkx2.1* is specifically involved in the development of rostral ventral forebrain including the pallidum and preoptic regions, but its function in the basal hypothalamus appears redundant with both *nkx2.4* genes. In contrast, *nkx2.4a* and *nkx2.4b* control aspects of basal hypothalamus development including the intermediate and caudal hypothalamus, where loss of *nkx2.4* activity is not fully compensated by *nkx2.1*.

## Author contributions

Wolfgang Driever and Martha Manoli designed the study. Martha Manoli performed all experiments and documentation. Martha Manoli and Wolfgang Driever carried out data analysis and wrote and edited the manuscript. Wolfgang Driever obtained funding and supervised the project.

### Conflict of interest statement

The authors declare that the research was conducted in the absence of any commercial or financial relationships that could be construed as a potential conflict of interest.
