# Supplementary material for: nkx2.1 and nkx2.4 genes function partially redundant during development of the zebrafish hypothalamus, preoptic region, and pallidum
Source: Front Neuroanat. 2014 Dec 2;8:145. doi: 10.3389/fnana.2014.00145 (PMC4251446; doi:10.3389/fnana.2014.00145)
Supplement: Supplementary file 1 [file Table1.DOC]

***nkx2.1* and *nkx2.4* genes function partially redundant during development of the zebrafish hypothalamus, preoptic region, and pallidum**

Martha Manoli and Wolfgang Driever

**Supplemental Table 1:**  Morpholino amounts injected in this study. Morpholinos as specified in Materials and Methods

|  | **SCMO** | **nkx2.4b TBMO** | **nkx2.4b(-2) TBMO** | **nkx2.1 TBMO** | **nkx2.4a SBMO** | **nkx2.4a TBMO** | **p53 MO** | **sum** |
| --- | --- | --- | --- | --- | --- | --- | --- | --- |
| 3C | 7.5ng |  |  |  |  |  | 2.5ng | 10ng |
| 3D |  | 2.5ng |  | 2.5ng | 2.5ng |  | 2.5ng | 10ng |
| 3E | 7.5ng |  |  |  |  |  | 2.5ng | 10ng |
| 3F |  | 2.5ng |  | 2.5ng | 2.5ng |  | 2.5ng | 10ng |
| 3G | 7.5ng |  |  |  |  |  | 2.5ng | 10ng |
| 3H |  | 2.5ng |  | 2.5ng | 2.5ng |  | 2.5ng | 10ng |
| 3I | 7.5ng |  |  |  |  |  | 2.5ng | 10ng |
| 3J |  | 2.5ng |  | 2.5ng | 2.5ng |  | 2.5ng | 10ng |
| 3K | 7.5ng |  |  |  |  |  | 2.5ng | 10ng |
| 3L |  | 2.5ng |  | 2.5ng | 2.5ng |  | 2.5ng | 10ng |
| 4A | 5ng |  |  |  |  |  |  | 5ng |
| 4B |  | 5ng |  |  |  |  |  | 5ng |
| 4C | 5ng |  |  |  |  |  |  | 5ng |
| 4D |  |  |  | 5ng |  |  |  | 5ng |
| 4E | 5ng |  |  |  |  |  |  | 5ng |
| 4F |  |  |  |  |  | 5ng |  | 5ng |
| 5A | 7.5ng |  |  |  |  |  | 2.5ng | 10ng |
| 5B |  |  |  | 7.5ng |  |  | 2.5ng | 10ng |
| 5C |  | 3.75ng |  |  | 3.75ng |  | 2.5ng | 10ng |
| 5D |  | 2.5ng |  | 2.5ng | 2.5ng |  | 2.5ng | 10ng |
| 5E | 7.5ng |  |  |  |  |  | 2.5ng | 10ng |
| 5F |  |  |  | 7.5ng |  |  | 2.5ng | 10ng |
| 5G |  | 3.75ng |  |  | 3.75ng |  | 2.5ng | 10ng |
| 5H |  | 2.5ng |  | 2.5ng | 2.5ng |  | 2.5ng | 10ng |
| 5I | 7.5ng |  |  |  |  |  | 2.5ng | 10ng |
| 5J |  |  |  | 7.5ng |  |  | 2.5ng | 10ng |
| 5K |  | 3.75ng |  |  | 3.75ng |  | 2.5ng | 10ng |
| 5L |  | 2.5ng |  | 2.5ng | 2.5ng |  | 2.5ng | 10ng |
| 5M | 7.5ng |  |  |  |  |  | 2.5ng | 10ng |
| 5N |  | 2.5ng |  | 2.5ng | 2.5ng |  | 2.5ng | 10ng |
| 5O | 7.5ng |  |  |  |  |  | 2.5ng | 10ng |
| 5P |  | 2.5ng |  | 2.5ng | 2.5ng |  | 2.5ng | 10ng |
| 5Q | 7.5ng |  |  |  |  |  | 2.5ng | 10ng |
| 5R |  | 2.5ng |  | 2.5ng | 2.5ng |  | 2.5ng | 10ng |
| 5S | 7.5ng |  |  |  |  |  | 2.5ng | 10ng |
|  | **SCMO** | **nkx2.4b TBMO** | **nkx2.4b(-2) TBMO** | **nkx2.1 TBMO** | **nkx2.4a SBMO** | **nkx2.4a TBMO** | **p53 MO** | **sum** |
| 5T |  | 2.5ng |  | 2.5ng | 2.5ng |  | 2.5ng | 10ng |
| 5U | 7.5ng |  |  |  |  |  | 2.5ng | 10ng |
| 5V |  | 2.5ng |  | 2.5ng | 2.5ng |  | 2.5ng | 10ng |
| 5W | 7.5ng |  |  |  |  |  | 2.5ng | 10ng |
| 5X |  | 2.5ng |  | 2.5ng | 2.5ng |  | 2.5ng | 10ng |
| 5Y | 7.5ng |  |  |  |  |  | 2.5ng | 10ng |
| 5Z |  | 3.75ng |  | 3.75ng |  |  | 2.5ng | 10ng |
| 5AA |  | 2.5ng |  | 2.5ng | 2.5ng |  | 2.5ng | 10ng |
| 6A | 7.5ng |  |  |  |  |  | 2.5ng | 10ng |
| 6B |  | 2.5ng |  | 2.5ng |  | 2.5ng | 2.5ng | 10ng |
| 6C | 7.5ng |  |  |  |  |  | 2.5ng | 10ng |
| 6D |  | 2.5ng |  | 2.5ng |  | 2.5ng | 2.5ng | 10ng |
| 7A | 7.5ng |  |  |  |  |  | 2.5ng | 10ng |
| 7B |  | 2.5ng |  | 2.5ng | 2.5ng |  | 2.5ng | 10ng |
| 7C | 7.5ng |  |  |  |  |  | 2.5ng | 10ng |
| 7D |  | 2.5ng |  | 2.5ng | 2.5ng |  | 2.5ng | 10ng |
| 7E | 7.5ng |  |  |  |  |  | 2.5ng | 10ng |
| 7F |  | 2.5ng |  | 2.5ng | 2.5ng |  | 2.5ng | 10ng |
| 8A | 7.5ng |  |  |  |  |  | 2.5ng | 10ng |
| 8B |  | 2.5ng |  | 2.5ng | 2.5ng |  | 2.5ng | 10ng |
| 8C | 7.5ng |  |  |  |  |  | 2.5ng | 10ng |
| 8D |  | 2.5ng |  | 2.5ng | 2.5ng |  | 2.5ng | 10ng |
| 8E | 7.5ng |  |  |  |  |  | 2.5ng | 10ng |
| 8F |  | 2.5ng |  | 2.5ng | 2.5ng |  | 2.5ng | 10ng |
| 8G | 7.5ng |  |  |  |  |  | 2.5ng | 10ng |
| 8H |  | 2.5ng |  | 2.5ng | 2.5ng |  | 2.5ng | 10ng |
